# Earth tectonics as seen by GOCE - Enhanced satellite gravity gradient imaging

**DOI:** 10.1038/s41598-018-34733-9

**Published:** 2018-11-05

**Authors:** Jörg Ebbing, Peter Haas, Fausto Ferraccioli, Folker Pappa, Wolfgang Szwillus, Johannes Bouman

**Affiliations:** 1 Institut für Geowissenschaften, Kiel University, Kiel, Germany; 2 British Antarctic Survey, Cambridge, UK; 3 Bundesamt für Kartographie und Geodäsie (BKG), Frankfurt am Main, Germany

**Keywords:** Geodynamics, Geophysics, Tectonics

## Abstract

Curvature components derived from satellite gravity gradients provide new global views of Earth’s structure. The satellite gravity gradients are based on the GOCE satellite mission and we illustrate by curvature images how the Earth is seen differently compared to seismic imaging. Tectonic domains with similar seismic characteristic can exhibit distinct differences in satellite gravity gradients maps, which points to differences in the lithospheric build-up. This is particularly apparent for the cratonic regions of the Earth. The comparisons demonstrate that the combination of seismological, and satellite gravity gradient imaging has significant potential to enhance our knowledge of Earth’s structure. In remote frontiers like the Antarctic continent, where even basic knowledge of lithospheric scale features remains incomplete, the curvature images help unveil the heterogeneity in lithospheric structure, e.g. between the composite East Antarctic Craton and the West Antarctic Rift System.

## Introduction

The GOCE (Gravity Field and Steady-State Ocean Circulation Explorer) satellite mission of the European Space Agency measured from 2009 to 2013 satellite gravity gradients at mean orbit heights of 255 and 225 km in the nominal and extended mission phases respectively. GOCE measurements fill a crucial gap in the spectral range between higher altitude missions, such as the GRACE mission, and near-surface measurements^1^. Since the end of the mission, a number of studies have exploited the GOCE datasets for different purposes. From the GOCE measurements that provided four gradient components with high accuracy in the measurement bandwidth, all six gradients have been computed in a Local North-Oriented Frame including GRACE information^2^. The LNOF gradients are a compromise between ease of access/application and maintaining as much as possible the original GOCE information. Keeping the initial gradient information is not necessarily the same as calculating gradients from global gravity field models, as in the latter the resolution of the gravity field model does affect the resolution of the gradients. This has been discussed and demonstrated in regional^3^ as well as global applications^2^. Applications include the use of the GOCE gravity data to estimate crustal thickness^4–7^, to model the structure of the lithosphere^8,9^ or to investigate deeper mantle sources^10^. Some of these studies demonstrated the additional sensitivity to the lateral sources distribution when using gravity gradients as compared to the conventional, vertical gravity field^2,11^. As typical for modelling and interpretation of the gravity field or its gradients, additional, constraining information is required, which is often provided from seismic or seismological models in form of a priori constraints. While the uncertainty in such models can affect the interpretation of gravity gradients^12^, the simultaneous use of all gradients makes one less prone to such errors^13^.

Still, the simultaneous interpretation of multiple gravity gradient components is particularly challenging. This is a well-known limitation e.g. in the interpretation of airborne gravity gradients for exploration^14^. Approaches like the use of third-order derivatives yield promising results^15^, but do not reduce the complexity in interpretation. Here, we adopt, therefore, recent advancements in the use of curvature components of the tensor that simplify the interpretation of gravity gradient datasets significantly and are directly making use of the second derivatives of the gravitational potential^16,17^. We apply these approaches to the GOCE satellite gravity data to derive the first global maps of the curvature components. Our curvature analysis is carried out after applying topographic and isostatic corrections, and this further enhances both lithospheric and intra-crustal sources (see the Methods section for details). Overall, we show that the curvature components of GOCE have significant potential for augmenting seismological imaging of the Earth’s lithosphere, e.g. to aid investigations of different cratonic regions, and for studying the lithosphere of the least understood continent on Earth, Antarctica.

## Results

### Curvature components at GOCE satellite altitude

**Figure 1:**
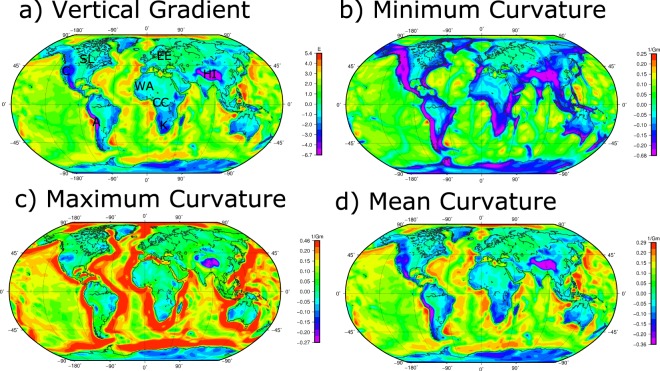
Global plots of curvature attributes. (**a**) Vertical Gradient (**b**) Minimum Curvature, (**c**) Maximum Curvature, (**d**) Mean Curvature. A: Andes, C: Cordillera, CC: Congo Craton, EE: East European Craton, HI: Himalaya, K: Kaapvaal Craton, SL: Slave Craton, WA: West African Craton.

Figure 1 shows the main curvature components and for comparison the vertical gravity gradient at satellite height of 225 km. In all components, the oceanic and continental domains are clearly differentiated. Within the continental areas the main tectonic elements, such as cratons and their boundaries and major orogenic belts are clearly imaged. The mean curvature shows the same features as the vertical gradient, as expected (see formula in the Methods section). The minimum and maximum curvature, however, illustrate the internal structure of the continents and oceans more clearly than the vertical gravity gradient. For example, internal differences within the continents are more readily apparent in the maximum curvature.

**Figure 2:**
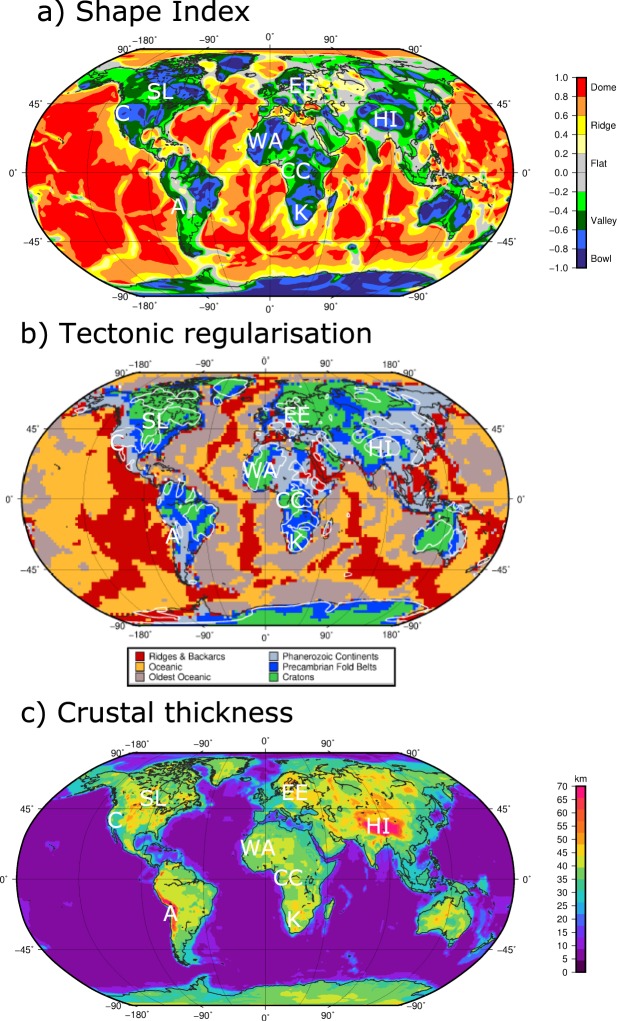
(**a**) Shape index from GOCE SSG. (**b**) Tectonic regularisation map of the Earth^18^. White contours correspond to the −0.6 value of the shape index. (**c**) Crustal thickness of Crust1.0^19^.

The more detailed structure within the continents is even better illustrated in the shape index shown in Fig. 2a. The values of the shape index can be expressed as dome- to bowl-like structures that the equipotential surface follows as an expression of a mass surplus or deficit at depth^17^. Bowl-shaped mass deficits correlate, in general, with orogenic belts and cratonic areas. For example, in the continental US, the cratonic core features a smaller mass deficit compared to the Cordillera in the west. This is expected, as for orogenic belts isostatic support in form of a crustal root is often observed (see also Himalaya). In contrast, mountain ranges associated with recent subduction zones like the Andes feature valley-like shape index structures, likely due to the mass surplus of the subducting slabs.

Notably, our new curvature products and especially the shape index vary quite significantly between individual cratons. Cratons are the oldest part of the continental crust and their seismological signatures are in general relatively similar, as expressed in the tectonic regularisation^18^ and crustal thickness maps^19^ (Fig. 2b,c).

A more detailed look at the crustal thickness shows that the cratons have similar, and despite the generally low topography, large crustal thickness. To explain this mismatch the concept of isopycnicity has been proposed in the late 1970 s^20^ and is still discussed today^21^. Isopycnicity explains the mismatch by a change in upper mantle composition and that the lithospheric mantle is lighter due to depletion of iron-rich elements. The seismic velocities mostly reflect the relatively cold temperature of the lithospheric mantle, less the depletion of the cratonic lithosphere^22^.

In the shape index, we can observe bowl- to valley- to flat-type areas over the cratons. While the West African and Kaapvaal Craton feature a bowl-shaped anomaly, the Congo Craton appears as a valley to almost flat-like area, and the Eastern European Platform is generally seen as a flat area. The differences in the shape index must be explained by density sources in the crust and/or the underlying uppermost mantle and are more sensitive to composition than temperature. The observed different shape index values imply substantial differences in the lithospheric build-up between such cratons. More indirectly, this behaviour has been previously discussed between the Slave and Kaapvaal Craton^23^.

Another example is the Congo Craton, for which dynamic support from the upper mantle has been proposed^24^. In this case, a lithospheric, viscous anomaly might lead to the apparent mass deficit compared to the surrounding cratons and modification of the old cratonic lithosphere by sub-lithospheric sources and processes has also been proposed recently^25^. The general high sensitivity to lithospheric sources and considerably lower sensitivity to deeper sources could point to processes like magmatic underplating modifying the crust. An example for this is the East European Craton, where the lowermost crust features unusual high seismic velocities and densities^26^. Such an anomalous lowermost crust is not observed for the Kaapvaal Craton and this is confirmed by the differences in the shape index.

GOCE-derived products have been previously used to discuss the tectonic setting of Africa, but using the free-air or Bouguer-anomalies and residuals thereof, but not directly the gradient products^27,28^. In these studies, a regression analysis was performed to remove the contributions to the gravity field by topography and isostasy, enhancing tectonic features in the residuals. Such analysis reveals more local scale features and allows to discuss the possible mass changes associated with magmatic intrusions, sedimentary basins and other features mostly within the upper crust. Our analysis differs from these studies as here we focus on the broader tectonic setting of the continents as curvature components at satellite height image the main building blocks of the continental lithosphere. This in turn helps us to identify differences in the lithospheric characteristics, which in combination with seismology, helps unravel the underlying causes for the differences between apparently similar continental domains, such as cratons.

### Curvature components over the Antarctic continent

The lithospheric structure of most continents is relatively well understood, at least in terms e.g. of basic knowledge regarding the extent of cratons, orogens, major rifts, and intracratonic basins, and the location of subduction and collision zones. However, comparable knowledge is still lacking for parts of the Antarctic continent, in spite of its global importance within the supercontinent cycle since the Archaean^29^ and the key influence that its lithosphere exerts on the overlying ice sheets^30^. The thick ice sheet cover and the remoteness of Antarctica make geological and geophysical investigations particularly challenging. Hence, Hdespite the large extent of recent aeromagnetic^31^ and aerogravity data coverage^32^, a continental-scale tectonic elements map that is required to aid global plate reconstructions^33^ remains to be defined.

**Figure 3:**
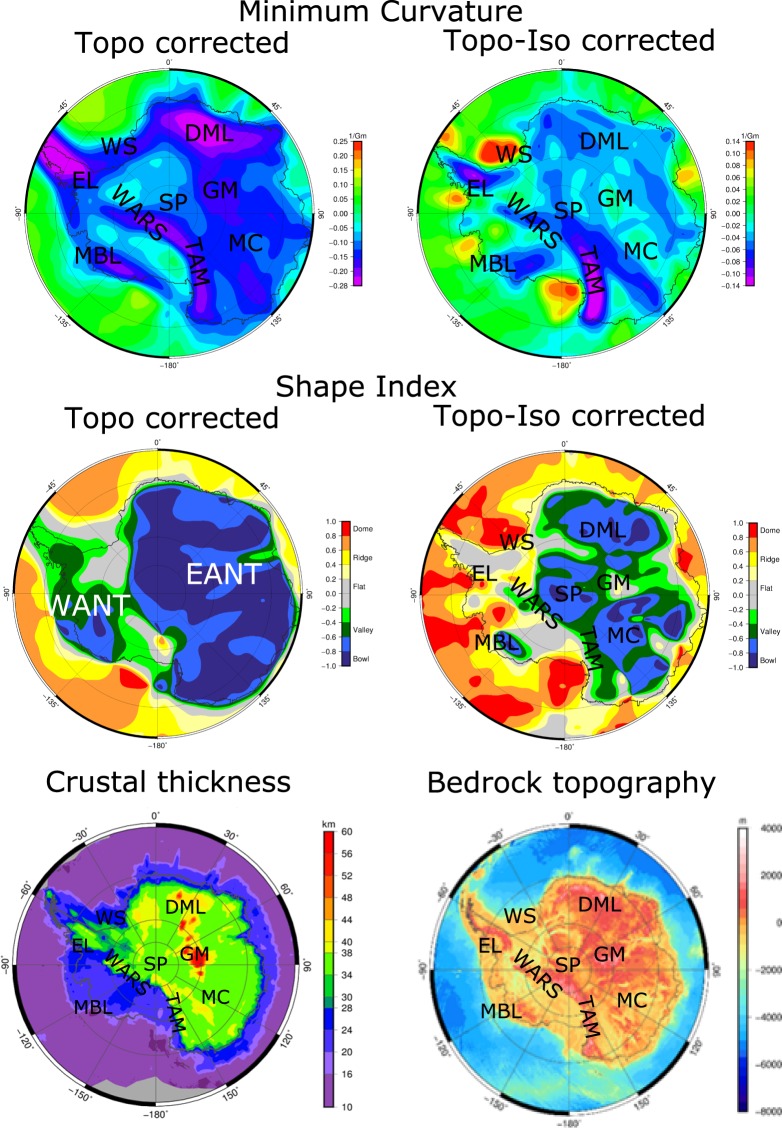
Comparison of GOCE products with Moho depth and bedrock topography for Antarctica. Left column: Minimum curvature and shape index after topographic correction. Right column: the same fields after additional isostatic correction. In the bottom: Moho depth^34^ and bedrock topography^48^. EANT = East Antarctica, WANT = West Antarctica, DML = Dronning Maud Land, EL = Ellsworth Land, GM = Gamburtsev Subglacial Mountains, MBL = Marie Byrd Land, MC = Mawson Craton, WS = Weddell Sea, SP = South Pole, WARS = West Antarctic Rift System, TAM = Transantarctic Mountains.

Here, we exploit our curvature products to aid ongoing investigations of the crustal and lithospheric architecture of Antarctica (Fig. 3). The most striking feature is the contrast between the thicker crust of the composite East Antarctic craton (40–60 km thick) and the thinner crust in West Antarctica (20–35 km thick), as imaged from both passive seismic^34^ and airborne gravity studies^35^. The Transantarctic Mountains formed along the lithospheric boundary between East and West Antarctica^36^, and despite being underlain by a small crustal root^37^, appear as a strong negative anomaly in the minimum curvature (Fig. 3). This suggests that an upper mantle thermal anomaly (leading to relatively lower densities) likely contributes to their isostatic support, as proposed from recent passive seismic investigations^38^.

To focus more on the internal structure of the continent, we discuss in the following isostatic corrected curvature components. In the isostatic corrected shape index (Fig. 3), the coastal area from Marie Byrd Land to Ellsworth Land features an alternation of positive and negative anomalies. Most notably, under Marie Byrd Land a bowl-like shape index is observed in the region of a proposed Cenozoic mantle plume^39^. While the shape index does not per se confirm the presence of a mantle plume, the anomaly supports the hypothesis for relatively lower density upper mantle beneath the Marie Byrd Land dome. We infer that this is likely linked to a thermal upper mantle anomaly independently proposed from seismic tomography^40^. Both the topographically corrected minimum and maximum curvature maps (Fig. 3 and Supplementary Material) reveal the continental-scale extent of the Cretaceous West Antarctic Rift System^41^ and the older Jurassic Weddell Sea Rift System^42^. However, narrower Cenozoic subglacial rift basins that are superimposed upon the broader region of extension in the West Antarctic Rift System, which are well resolved by airborne gravity, are not imaged by the satellite gravity data due to its spatial resolution (~80 km half-wavelength).

The interior of East Antarctica is thought, based on aeromagnetic and satellite magnetic interpretation, to be a mosaic of Precambrian cratonic provinces and orogenic belts of ill-constrained and yet hotly debated age and origin^29,43^. In the curvature products (and especially in the topographic and isostatic corrected shape index) interior East Antarctica appears to include at least three major heterogeneous lithospheric domains. One correlates with the Mawson Craton, which included also large parts of southern Australia prior to Gondwana break-up^44^, while the second corresponds to the region of the inferred Tonian Oceanic Arc Superterrane, in the interior of Dronning Maud Land^45^. These domains appear to be separated by the region of the Gamburtsev Subglacial Mountains, where the crust is up to 60 km thick, and an orogenic belt of inferred ca. 1 Ga^43^ or ca 550 Ma age^34^ has been proposed. The origin of the third domain, apparently lying between the Weddell Sea and South Pole, and its relation with the Mawson Craton remains unclear. This poorly explored region includes the so-called Polar Gap south of 83°S, where GOCE data are not available (due to the inclination of the satellite orbit) and hence lower resolution GRACE data are used instead. These three distinct domains are not apparent in currently available seismic tomography^34^ and represent an important new element to study Antarctica in relation to global plate tectonic reconstructions, both before and after the break-up of Gondwana (see Supplementary Material for an illustration).

## Conclusions and Outlook

Curvature products derived from GOCE data provide a new tool to help distinguish different lithospheric domains and their tectonic boundaries, at both continental and global scale. By revealing differences and similarities with respect to seismological results, our study shows the potential for further integrated analysis, especially in trying to decipher the build-up of the cratonic regions of the Earth. This goes beyond earlier studies that used the GOCE derived products to discuss tectonic domains and the internal crustal structure. Although these studies were able to identify tectonic structures, our analysis indicates that the characteristics of the lithosphere can differ even in apparently similar tectonic regimes. They can show quite different characteristics in the gravity gradients, while the seismic velocity structure is similar. Geochemical and petrological information is certainly a key to help understand these differences, but there are still intrinsic limitations due the paucity of available xenolith samples^46^.

In our current contribution, the analysis remains qualitative, but to advance towards a better understanding of the nature of the crust and mantle, the specific characteristics of the satellite gravity gradients should be exploited further. Potential theory demands that the internal consistency of the gradients has to be adhered. As these have different sensitivities to the source geometry, that can be used for example in probabilistic joint inversion schemes. While inversion of gravity gradients does not overcome the limitations of non-uniqueness, it will certainly limit the solution space.

Due to their resolution of 80 km, the satellite gravity gradients provide a tool to link global and large-scale regional studies in a more consistent manner. Such models based on the integration of different geophysical observables are a necessary step before advancing into detailed, local interpretation for which higher-resolution data as available from aerogeophysical or terrestrial measurements.

For Antarctica, which still stands out as the least understood continent on Earth, it is clear that the new GOCE curvature and shape index products have considerable potential towards further elucidating the heterogeneity in lithospheric architecture beneath the Antarctic ice sheets. The satellite gravity curvature and shape index products for Antarctica and other continents can be incorporated in plate reconstructions. This can help towards re-evaluating both the similarities and differences in lithospheric structure and sub-lithospheric processes between formerly adjacent continents, prior and after supercontinent break-up.

Overall, we emphasise that satellite data such as from the GOCE mission, provide novel datasets with a global homogenous coverage that can significantly advance our understanding of the Earth structure and tectonic setting.

## Methods

### GOCE data and corrections

The satellite gravity gradients (SGG) at satellite altitude of the GOCE satellite mission are given for gradient grids at 225 km above the Earth’s surface in a local north-oriented reference frame, NWU (North, West, Up), which is the convention adopted for GOCE^2^.

**Figure 4:**
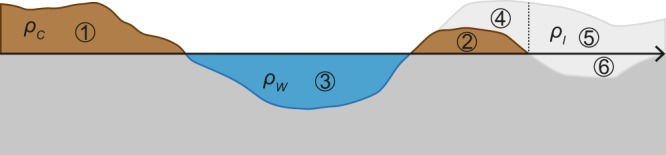
Scheme for a topographic mass correction. Masses 1, 2, 4, and 5 are considered with their absolute density, while masses 3 and 6 are considered relative to a normal density of 2670 kg m^−3^.

We performed a topographic correction using the ETOPO1^47^, supplemented by Bedmap2^48^ for the Antarctic region. Both datasets contain information on topography, bathymetry, and ice. Subsequently, rock, water, and ice masses are separately modelled with respective densities of 2670 kg m^−3^, 1030 kg m^−3^, and 917 kg m^−3^ (Fig. 4).

To evaluate the signal content in the SGG, we also calculated the effect of the isostatic compensation and removed this effect from the topographic reduced fields^49^. For the onshore areas, an Airy-type crustal isostatic model is used, which is a sufficient approximation for long wavelengths. In oceanic areas, lateral variations of density exist in the oceanic lithosphere, which we approximate by assuming Pratt-type isostasy.

Our reference model has no topography and a crustal thickness of *c* = 30 km. The applied density contrast between crust and mantle is 400 kg m^−3^ ^49^.

All calculations are done with tesseroids^50^. A tesseroid is a volume element defined on a sphere. When a density is assigned to a tesseroid, one can compute its gravitational potential, gravity and gravity gradients.

### Curvature

Curvature describes how much a line deviates from being straight or a surface from being flat^17^. Unlike the first-order derivative methods for delineation of lineaments, curvature contains the added dimension of shape. For a 3D surface, curvature is also independent of the surface orientation.

From theory, many curvature attributes can be computed. Curvature has been widely used to interpret geophysical data after the introduction of 11 curvature attributes, which have been selected based on their applicability to seismic interpretation^51^. Only a small number are independent from each other and rarely more than the original 11 attributes and the differential curvature are applied to geophysical data^17^. The full mathematical background, tests with synthetic data and an evaluation of these attributes for airborne gravity gradients can be found in^17^.

When curvature is used to interpret gravity anomalies, we try to delineate geometric information of subsurface structures from an observed non-geometric quantity.

The mean curvature is found with the following expression:

$${{\rm{K}}}_{{\rm{mean}}}=-\,\frac{{{\rm{T}}}_{{\rm{NN}}}+{{\rm{T}}}_{{\rm{WW}}}}{2{\rm{g}}}=\frac{{T}_{UU}}{2g}$$

The mean curvature is a function of two gradient components T_NN_ and T_WW_ in derivation. It can be expressed as a function of T_UU_ as well because of Laplace’s equation.

The second attribute is Gauss curvature. With the same procedure, this is defined as:

$${{\rm{K}}}_{{\rm{gauss}}}=-\,\frac{{{\rm{T}}}_{{\rm{NN}}}{{\rm{T}}}_{{\rm{WW}}}-{{{\rm{T}}}_{{\rm{NW}}}}^{2}}{{{\rm{g}}}^{2}}$$

The Gaussian curvature is the product of minimum and maximum curvatures and often exhibits rapid sign changes. These two attributes are not particularly useful^17^, but they can be used to compute many of the other components. Two of those are minimum and maximum curvature, which can be expressed as:

$$\begin{array}{rcl}{{\rm{K}}}_{{\rm{\max }}} & = & {{\rm{K}}}_{{\rm{mean}}}+\sqrt{{{{\rm{K}}}_{{\rm{mean}}}}^{2}-{{{\rm{K}}}_{{\rm{gauss}}}}^{2}}\\ {{\rm{K}}}_{{\rm{\min }}} & = & {{\rm{K}}}_{{\rm{mean}}}-\sqrt{{{{\rm{K}}}_{{\rm{mean}}}}^{2}-{{{\rm{K}}}_{{\rm{gauss}}}}^{2}}\end{array}$$

Or in terms of gravity gradients:

$$\begin{array}{rcl}{{\rm{K}}}_{{\rm{\max }}} & = & \frac{{{\rm{T}}}_{{\rm{NN}}}+{{\rm{T}}}_{{\rm{WW}}}-2\sqrt{{{{\rm{T}}}_{{\rm{NW}}}}^{2}+{{{\rm{T}}}_{{\rm{UV}}}}^{2}}}{2{\rm{g}}}\\ {{\rm{K}}}_{{\rm{\min }}} & = & \frac{{{\rm{T}}}_{{\rm{NN}}}+{{\rm{T}}}_{{\rm{WW}}}+2\sqrt{{{{\rm{T}}}_{{\rm{NW}}}}^{2}+{{{\rm{T}}}_{{\rm{UV}}}}^{2}}}{2{\rm{g}}}\end{array}$$

with

$${{\rm{T}}}_{{\rm{UV}}}=({{\rm{T}}}_{{\rm{NN}}}-{{\rm{T}}}_{{\rm{WW}}})/2$$

Maximum and minimum curvature can be combined in a different way to compute the shape index:

$${\rm{SI}}=\frac{2}{{\rm{\pi }}}{\tan }^{-1}\frac{-{{\rm{T}}}_{{\rm{NN}}}-{{\rm{T}}}_{{\rm{WW}}}}{2\sqrt{{{{\rm{T}}}_{{\rm{NW}}}}^{2}+{{{\rm{T}}}_{{\rm{UV}}}}^{2}}}$$

This shape index is a quantitative description of the shape of the local morphology and is not affected by the absolute magnitude of curvature.

The shape index that allows a more quantitative shape description and its amplitude is always within +/−1: bowl (−1), valley (−0.5), flat (0.0), ridge (−0.5), and dome (−1). These characteristic numbers are derived and work perfectly for geometric surfaces^51,52^.

Two remarks have to be made: First, the gravity gradients are not absolute values, but they are gravity gradient anomalies. Hence the term curvature should be substituted by a definition as ‘curvature perturbation’ or ‘anomaly of an equipotential surface’^17^. To avoid ambiguity, the term curvature is used here in the sense of ‚ a curvature perturbation’.

As a second point, some of the curvature attributes contain the gravity g in the denominator. When using the gravity disturbance, this would result in singularity for a gravity anomaly of zero or small values. To prevent this, g has to be weighted with a positive scaling summand. The value of the summand is not that relevant, as long as it is greater than the maximum topographic corrected gravity disturbance. If g changes, the qualitative value of the curvature will also change, but the amplitude of the resulting curvature remains unaffected. For convenience, the scaling summand is the maximum value of absolute gravity at satellite height, which is 914180 mGal (9.1418 *m s*−2).

### Tectonic regularisation and crustal thickness map

The tectonic regularisation map in Fig. 2 was computed by the authors^18^ from the model SL2013sv using the *k-means* clustering. For this, they utilized the very large phase- and group-velocity dataset created in the course of the construction of their global tomographic model SL2013sv^22^, as well as the model itself. SL2013sv offers increased resolution globally, approaching that of regional-scale studies. The vertical-component data set used to compute consists of almost three quarters of a million waveform fits, from which more than half a million of the most mutually-consistent seismograms were selected to constrain the model.

The crustal thickness map in Fig. 2 is from the global model Crust1.0^19^ with 1 × 1 degree lateral resolution. The model is based on an extensive seismic catalogue, but uses a crustal age map^53^ to predict crustal thickness in areas not well covered by seismic measurements.

### Plate-tectonic illustration in supplementary material

The movie in the supplementary material provides an example for the last 200 Myr of plate tectonics centred over Antarctica. The movie illustrates by the use of the topographic reduced shape index the link between Antarctica and the adjacent continents. The plate-tectonics illustration was done in GPlates (https://www.gplates.org/).

## Electronic supplementary material

Supplementary material

## Data Availability

The curvature components data are available under https://www.3dearth.uni-kiel.de.
