# Author Correction: Earth tectonics as seen by GOCE - Enhanced satellite gravity gradient imaging

**DOI:** 10.1038/s41598-019-48629-9

**Published:** 2019-08-28

**Authors:** Jörg Ebbing, Peter Haas, Fausto Ferraccioli, Folker Pappa, Wolfgang Szwillus, Johannes Bouman

**Affiliations:** 1 Institut für Geowissenschaften, Kiel University, Kiel, Germany; 2 British Antarctic Survey, Cambridge, UK; 3 Bundesamt für Kartographie und Geodäsie (BKG), Frankfurt am Main, Germany

Correction to: *Scientific Reports* 10.1038/s41598-018-34733-9, published online 05 November 2018

This Article contains errors in Figure 3.

The top two images in the first column that show the minimum curvature and shape index after topographic correction, and the top two images in the second column that show the same fields after additional isostatic correction, have all been incompletely plotted.

The complete correct Figure 3 appears below as Figure 1.

**Figure 1:**
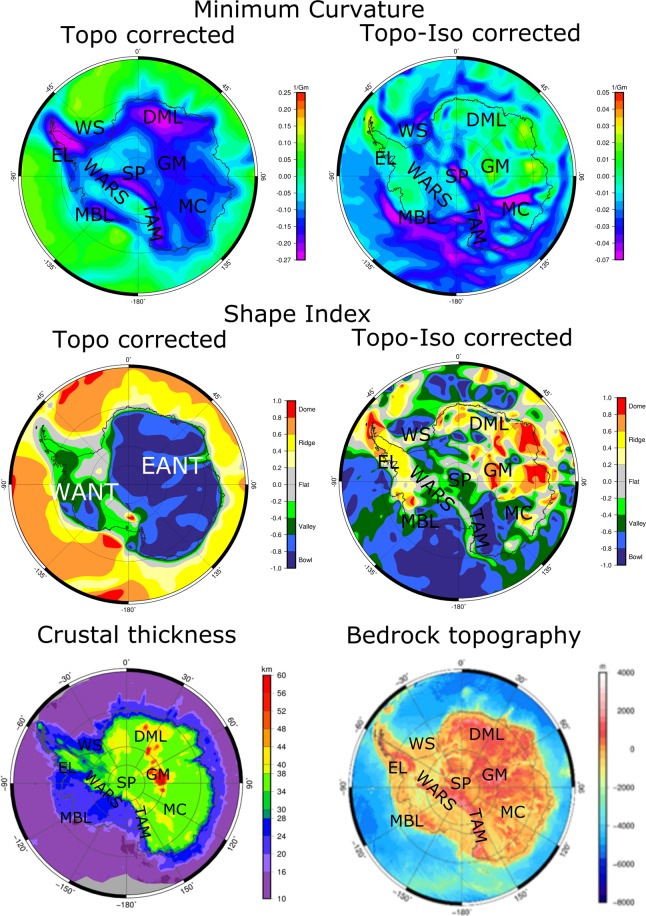
Comparison of GOCE products with Moho depth and bedrock topography for Antarctica. Left column: Minimum curvature and shape index after topographic correction. Right column: the same fields after additional isostatic correction. In the bottom: Moho depth^34^ and bedrock topography^48^. EANT = East Antarctica, WANT = West Antarctica, DML = Dronning Maud Land, EL = Ellsworth Land, GM = Gamburtsev Subglacial Mountains, MBL = Marie Byrd Land, MC = Mawson Craton, WS = Weddell Sea, SP = South Pole, WARS = West Antarctic Rift System, TAM = Transantarctic Mountains.

